# Effects of virtual reality natural experiences on factory workers’ psychological and physiological stress

**DOI:** 10.3389/fpsyg.2023.993143

**Published:** 2023-03-06

**Authors:** Mu-Hsing Ho, Meng-Shin Wu, Hsin-Yen Yen

**Affiliations:** ^1^School of Nursing, LKS Faculty of Medicine, The University of Hong Kong, Pokfulam, Hong Kong SAR, China; ^2^School of Gerontology and Long-Term Care, College of Nursing, Taipei Medical University, Taipei, Taiwan

**Keywords:** green space, heart rate variability, immersion, mental health, occupational health, virtual reality

## Abstract

**Introduction:**

Manufacturing facilities and factories are stressful work environments. Interventions to improve factory workers’ stress is necessary to promote occupational health. This study aimed to examine the effects of virtual reality natural experiences on furniture factory employees’ psychological and physiological stress.

**Methods:**

A single-blinded, non-randomised quasi-experimental study was conducted between July and December 2021. Factory workers were recruited from two factories, and all participants at a given factory were assigned to either an experimental group or a comparison group. The intervention was conducted in a clean conference room once a week for 12 weeks during the worker’s break time. The experimental group received virtual reality natural experiences consisting of 30-minute nature-based 360° videos which were played in a headset. The generalised estimating equations were performed for the statistical analyses.

**Results:**

In total, 35 participants completed the intervention. As to psychological stress, the experimental group showed improvements in distress, depression, and anxiety, and a positive affect after the intervention compared to the comparison group. As to physiological stress, the experimental group showed improvements in indicators of heart rate variability compared to the comparison group, including standard deviations of all normal-to-normal intervals, low-frequency power, and high-frequency power.

**Discussion:**

Virtual reality is an innovative platform to bring the natural environment into an indoor environment to create similar health effects.

## 1. Introduction

Occupational stress is a well-known issue worldwide that influences both developed and developing countries. Workplace stress occurs when work-related demands surpass a worker’s capacity to manage them (World Health Organization, 2020). Moreover, globalization and dramatic changes have had direct impacts on the variety of work in terms of technological developments, higher job demands, and workloads. Also, aging populations and the demographic and systemic structure of the workforce, such as a poor work-life balance, job insecurity, and precarious employment, have resulted in a significant occupational stress epidemic worldwide (Sorensen et al., 2021). Work stress is particularly important and significantly impacts individuals and organizations. Workplace stress causes a variety of ailments, including cardiovascular and metabolic disorders, psychological issues, musculoskeletal discomfort, reproductive issues, and occupational injuries, and also leads to a poor quality of life (Mohamed et al., 2022). These health issues are related to increased absenteeism and presenteeism, as well as decreased motivation, contentment, and commitment. These can produce increases in employee turnover and a desire to resign, resulting in low business productivity and increased medical, healthcare, and social welfare expenditures (Asplund et al., 2022; Mohamed et al., 2022; Sznajder et al., 2022).

Manufacturing is not exempt from stressful work environments. Workers in the manufacturing industry become stressed as a result of high job expectations, lengthy and irregular working hours, and tough work shift patterns in order to reach production objectives and maintain customer satisfaction. Constant work stress and pressure result in both physical and mental exhaustion, a lack of work-life balance, and decreased employee productivity (Bhui et al., 2016; Bolliger et al., 2022; Kim and Jung, 2022). Hopelessness, not feeling useful, and feeling depressed in the work environment are considered factors associated with symptoms of work-related stress among factory workers (Sznajder et al., 2022), highlighting the need for interventions to alleviate poor mental health symptoms among workers in high-pressure occupational environments.

Nature-based interventions have been studied and are considered effective strategies for alleviating stress and mental health illnesses (Picton et al., 2020; Coventry et al., 2021). Nature-based interventions provide individuals with an opportunity to explore their relationship with nature in terms of connecting to and being impacted by the natural environment to reduce negative mental health issues (Hartig et al., 2014; Owens and Bunce, 2022). However, infusing the natural environment in the workplace is challenging due to urbanization, and people who live and work in urban areas have very limited opportunities to connect with nature. A scarcity of research has been undertaken to implement nature-based interventions and build a natural environment in the factory workplace.

Virtual reality (VR) is becoming an increasingly popular technology, and a growing body of research has demonstrated the effect of using VR as a tool to enable engagement with natural environments (Li et al., 2021; Adhyaru and Kemp, 2022; Spangenberger et al., 2022). Several nature videos and applications can also be easily accessed and applied as VR technology (Adhyaru and Kemp, 2022; Calogiuri et al., 2022). Implementing a natural environment using VR is a novel approach and likely to produce psycho-physiological benefits by bringing nature into an indoor environment (Browning et al., 2019). A previous evidence-based study revealed that using VR natural experiences had positive impacts on psychological stress in terms of mood, anxiety, perceived stress, and physiological stress such as the heart rate (HR) (Adhyaru and Kemp, 2022). However, few investigations have been conducted into the impacts of VR natural experiences on biofeedback and physiological stress. A more in-depth discussion and evidence are required of the physiological changes such as the autonomic nervous system (ANS) and blood pressure (BP) measurements through VR natural experiences (Lüddecke and Felnhofer, 2022). Moreover, research touched on both psychological and physiological stress-related outcome is scarce. VR natural experiences can be considered as a simulation-based intervention contributing to mental-state attribution through the simulation of perception. According to the simulation theory, activity in sensory cortex that resembles the perception of external stimuli can be elicited from other parts of the brain. Particularly from a simulation-based intervention, imagining, hearing, or feeling something is essentially the same as actually seeing, hearing, or feeling it (Hesslow, 2012). Therefore, VR natural experiences intervention which brought a natural environment into a workplace has a great potential and contribution to psychological and physiological stress improvement. However, research on utilizing VR natural experience on alleviating occupational stress are limited. More empirical studies on investigating the effect of VR natural experience on occupational stress are warranted. Thus, the main purpose of this study was to explore the effects of VR natural experiences on furniture factory employees’ stress. Using an innovative intervention of VR natural experiences during their break time, the factory workers in the experimental group were expected to show improvements in their psychological and physiological stress compared to the comparison group. Psychological stress-related outcomes included distress, depression, anxiety, somatization, positive and negative affects, and perceived stress. Physiological stress-related outcomes included BP and HR variability (HRV).

## 2. Materials and methods

### 2.1. Study design

This was a single-blinded, two-armed non-randomized, quasi-experimental study conducted from July to December 2021. Participants were recruited from two furniture factories by convenience sampling in southern Taiwan. The supervisors of the two factories were contacted by the principal investigator, and the oral consent was obtained to invite eligible workers in the factories. Then, a researcher explained the aim and procedure of the study to all workers. All workers were required to sign an informed consent form before data collection and the intervention. The workers in the two factories were either assigned to an experimental group (VR group) and a comparison group by drawing. The minimal sample size was 34 which was calculated by medium effect sizes *via* G^*^power software (Faul et al., 2007). Each factory recruited 21 participants who were either in the same experimental or comparison group. Participants were not aware of the other group. Ethical approval for this study was obtained from the Taiwan Medical University-Joint Institutional Review Board (N202103114). This study was conducted in accordance with the principles of the Declaration of Helsinki.

### 2.2. Participants

**Figure 1:**
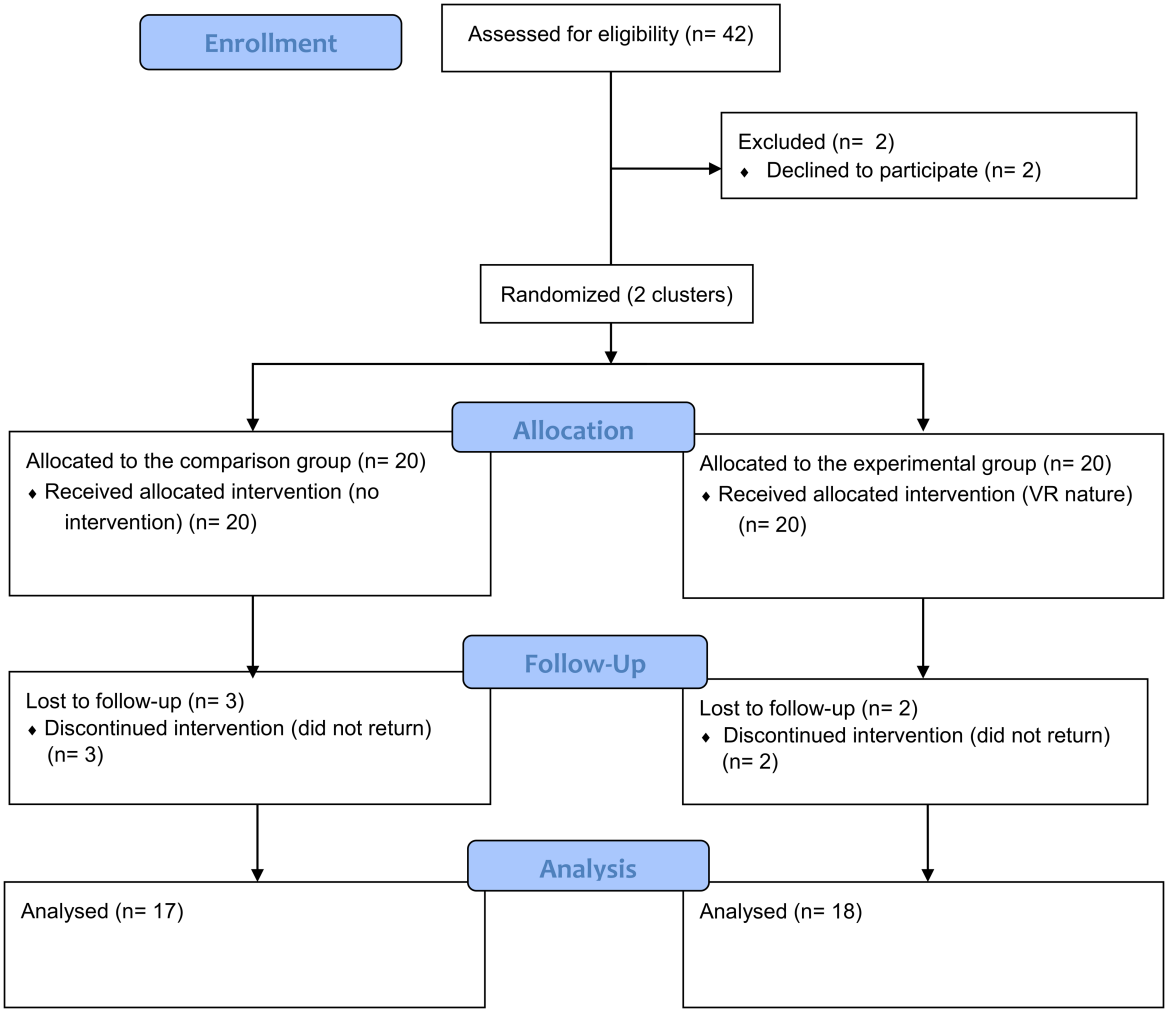
Flow diagram detailing the progress of enrolment to analysis.

Participants were factory workers who had break time in the afternoon. The inclusion criteria were participants (1) aged 20–60 years, (2) who worked on the production line, (3) who had no visual or hearing impairment, and (4) who had no serious health problems, mental illness, or disability that might influence the experiment and outcomes. The exclusion criteria were participants (1) who had gone to a natural environment for recreation in the past year, (2) who went to parks or green spaces weekly, (3) who had experiences in using any VR devices in the past year, and (4) who experienced serious VR sickness. The study flow is illustrated in Figure 1.

### 2.3. The intervention

**Figure 2:**
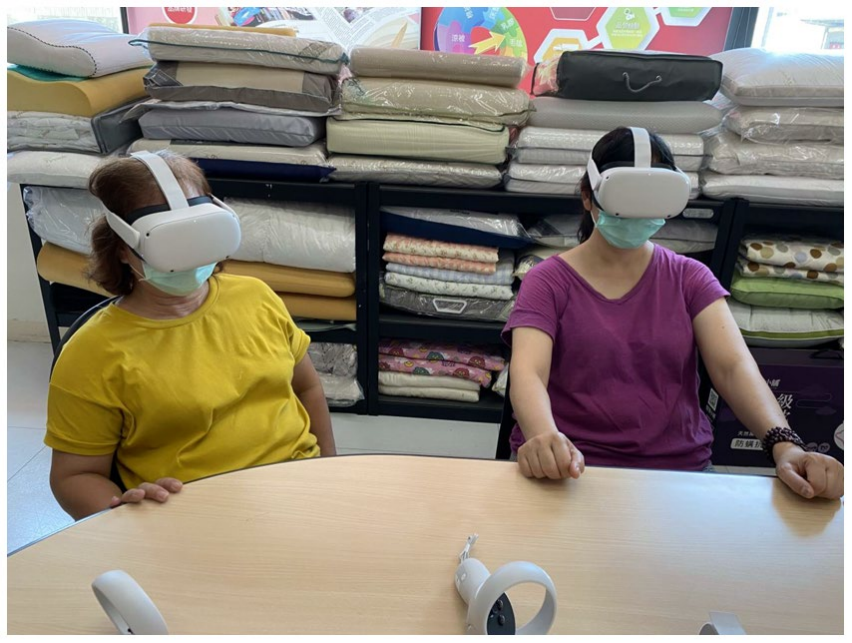
Photo of the virtual reality (VR) natural experience intervention.

The intervention was conducted once a week for 12 weeks. To not interrupt the working time, the intervention in the experimental VR group was conducted during workers’ break time. Five participants with the same break time schedule were grouped together. The group received the VR intervention in a clean conference room at the same time. The conference room was free of interference from any external visual or auditory stimulation. Participants in the experimental group were required to sit in a chair and wear a VR headset (Oculus Quest 2, META, United States) to watch 360° videos for 30 min (Figure 2). Based on the coronavirus disease 2019 (COVID-19) policy at that time, participants were also required to wear a face mask in indoor environments.

Nature-based VR videos were pre-recorded in a 360° format, including such areas as parks, hiking trails, forest paths, and bikeways (Supplementary Figures S1, S2). All videos were recorded on a sunny day in the afternoon. A different 30 min nature-based video was played in the VR headset every week. During the session, participants could freely move the direction of their head to watch the video from various angles. Participants were asked not to talk to each other during the session. A trained college student supervised every session. When using the VR headset, if a participant felt a little uncomfortable, they were told to temporarily close their eyes and then open their eyes again. If the uncomfortable sensation continued, they were told to stop the session. The comparison group received no interventions for 12 weeks. Participants in the comparison group were free to do any activities of their choosing during their afternoon break time.

### 2.4. Measures

Measurements were conducted in the conference room once before and once after the 12 week intervention by a trained college student. Stress-related outcomes were measured by self-reported questionnaires, a sphygmomanometer, and an HRV analyzer. Participants’ background information was collected in self-reported, structured questionnaires, including age, gender, education level, marital status, main job content in the factory, alcohol use, smoking, and chronic diseases.

Psychological measures. The Four-Dimensional Symptom Questionnaire (4DSQ) measures four common mental health problems: distress, anxiety, depression, and somatization. In total, 50 items were measured on a five-point scale. A higher score indicates worse symptoms. The 4DSQ previously presented good content validity, criterion-related validity, and construct validity (Terluin et al., 2016). Cronbach’s α was 0.802 in this study. The Positive and Negative Affect Scales (PANAS) were used for measuring participants’ emotions in two dimensions, including positive and negative affects. In total, 50 items were measured on a five-point scale. A higher score indicates a higher perceived affective status. The PANAS have good reliability and construct validity (Watson et al., 1988). The Perceived Stress Scale (PSS) was used for a self-evaluation of stress in the past month. In total, 10 items were measured on a five-point scale. A higher score indicates higher perceived stress. The PSS previously had good reliability, construct validity, and criterion-related validity (Cohen et al., 1983). Cronbach’s *α* was 0.903 in this study.

Physiological measures. A BP monitor (HEM-7310, OMRON, Japan) was used to measure participants’ systolic BP (SBP) and diastolic BP (DBP). The BP monitor was validated European Society of Hypertension International Protocol. by A handheld electrocardiogram (ECG) Monitor (8Z11, Wegene Technology, Taiwan) was used for ECG signal acquisition, storage, and processing of resting HRV and the ANS with the good validity (Tseng et al., 2020). Participants were requested to sit still for 5 min to record short-term HRV. After the algorithm, the selective parameters of HRV included the HR, a standard deviation (SD) of all normal-to-normal intervals (SDNN), total power (TP, 0–0.5 Hz), low-frequency (LF) power (0.04–0.15 Hz), high-frequency (HF) power (0.15–0.40 Hz), and the ratio of LF to HF (LF/HF). Participants with a higher SDNN and LF/HF were more likely to have better ANS and lower stress, anxiety, and depression (Malik et al., 1996).

### 2.5. Statistical analysis

Descriptive analyzes used the frequency and percentage for categorized variables and the mean and SD of continuous variables. Chi-square tests were performed to compare participants’ backgrounds between the experimental and comparison groups. Cramer’s V was calculated for effect sizes of the Chi-squared tests. Independent t-tests were performed to compare differences in participants’ age and stress-related outcomes of psychological and physiological measures at the baseline. Cohen’s d was calculated for effect sizes of the independent t-tests. The natural logarithms (Ln) of HRV data (TP, LF, HF, and LF/HF) were calculated for further analyzes. Analyzes were performed based on intention-to-treat principle. ITT approach provides unbiased comparisons among the treatment groups and this technique was done to avoid the effects of dropout, which the number of participants after group allocation was included in the final analysis (i.e., VR group *N* = 20 and Comparison group *N* = 20). Generalized estimating equations (GEEs) were performed to analyze the effect of group, time, and group-by-time interactions on stress-related outcomes. The GEEs were adjusted for participants’ age and the score at the baseline. SPSS 18.0 (SPSS, United States) was used for all statistical analyzes.

## 3. Results

### 3.1. Participants’ backgrounds

Table 1 reveals the participants’ backgrounds. No significant differences were found in gender, smoking behavior, or chronic diseases between the VR and comparison groups. However, participants’ age, education levels, marital status, and job content exhibited significant differences between the two groups. Therefore, the significant continuous variable (age) of participants’ backgrounds was adjusted for in the subsequent GEE analysis. In total, 18 participants in the VR group and 17 participants in the comparison group completed the intervention.

**Table 1:** Participants’ backgrounds.

Parameter		VR group (*N* = 20)		Comparison group (*N* = 20)		*x* ^2^	*p*	V^1^
		*n*	(%)	*n*	(%)			
Gender	Male	7	(36.84%)	12	(60.00%)	2.09	0.148	0.232
	Female	12	(63.16%)	8	(40.00%)			
Educational level	<high school	13	(81.25%)	6	(33.33%)	7.89	0.005	0.482
	>college	3	(18.75%)	12	(66.67%)			
Marital status	Single/divorced	3	(15.79%)	10	(50.00%)	5.13	0.023	0.363
	Married	16	(84.21%)	10	(50.00%)			
Job content	Tailor	5	(26.32%)	5	(25.00%)	8.62	0.013	0.470
	Sewing	8	(42.11%)	1	(5.00%)			
	Others	6	(31.58%)	14	(70.00%)			
Alcohol use	No	13	(86.67%)	10	(50.00%)	5.12	0.024	0.382
	Yes	2	(13.33%)	10	(50.00%)			
Smoking	No	3	(16.67%)	2	(10.00%)	0.37	0.544	0.098
	Yes	15	(83.33%)	18	(90.00%)			
Chronic diseases	No	14	(73.68%)	17	(85.00%)	0.77	0.382	0.140
	Yes	5	(26.32%)	3	(15.00%)			

^1^Cramer’s *V* for effect size. VR, virtual reality.

### 3.2. Stress-related outcomes at the baseline

Table 2 demonstrates participants’ stress-related outcomes at the baseline. Negative affect (*p* = 0.043) and SBP (*p* = 0.043) were found to significantly differ between the VR and comparison groups. No other variables of psychological or physiological measures were found to significantly differ between the VR and comparison groups.

**Table 2:** Participants’ stress-related outcomes at the baseline.

Variable		VR group (*N* = 20)		Comparison group (*N* = 20)		*t*	*p*	*d*
		Mean	(SD)	Mean	(SD)			
Age (years)		55.21	(7.71)	36.10	(11.12)	6.27	<0.001	1.998
Psychological measures	Distress	23.24	(6.51)	23.05	(4.43)	0.10	0.919	0.033
	Depression	8.12	(3.30)	6.70	(1.42)	1.65	0.114	0.559
	Anxiety	15.24	(4.52)	14.80	(3.65)	0.32	0.748	0.106
	Somatization	25.29	(8.07)	24.10	(5.31)	0.54	0.593	0.175
	Positive affect	2.71	(1.06)	2.68	(0.70)	0.09	0.930	0.028
	Negative affect	2.26	(0.85)	1.76	(0.64)	2.09	0.043	0.668
	Perceived stress	25.63	(4.57)	26.40	(4.84)	−0.51	0.614	0.163
Physiological measures	SBP (mmHg)	132.90	(15.73)	118.63	(16.09)	2.80	0.008	0.897
	DBP (mmHg)	80.35	(11.72)	73.05	(10.61)	2.04	0.050	0.653
	HR (bpm)	75.20	(10.13)	80.00	(13.73)	−1.25	0.220	0.398
	SDNN (ms)	39.02	(17.16)	44.75	(19.85)	−0.97	0.341	0.309
	TP [Ln (ms^2^)]	6.94	(0.99)	7.32	(0.95)	−1.22	0.229	0.392
	LF [Ln (ms^2^)]	5.58	(1.31)	6.06	(0.84)	−1.36	0.181	0.439
	HF [Ln (ms^2^)]	4.97	(1.35)	5.46	(1.23)	−1.17	0.249	0.376
	LF/HF [Ln (ratio)]	0.61	(0.70)	0.61	(0.79)	0.01	0.990	0.004

Cohen’s *d* for effect size; VR, virtual reality; SD, standard deviation; SBP, systolic blood pressure; DBP, diastolic blood pressure; HR, heart rate; SDNN, standard deviation of all normal-to-normal intervals; TP, total power (0–0.5 Hz); LF, low-frequency power (0.04–0.15 Hz); HF, high-frequency power (0.15–0.40 Hz); LF/HF, the ratio of low frequency to high frequency.

### 3.3. Outcomes

Table 3 demonstrates the GEE-adjusted model which indicates the effects of group, time, and group-by-time interactions on stress-related outcomes, and the model was adjusted for participants’ age and the outcome score at the baseline. For psychological measures, significant group effects (*p* = 0.021) and group-by-time interactions (*p* = 0.015) were found for distress. Significant group effects (*p* = 0.039) and group-by-time interactions (*p* = 0.042) were also found for anxiety. Significant group effects were found for depression (*p* = 0.005) and positive affect (*p* = 0.035). Mean differences indicated that distress, depression, anxiety, and positive affect in the VR group improved after the intervention compared to the comparison group. In contrast, somatization, negative affect, and perceived stress revealed no significant effects.

**Table 3:** Results of the generalized estimating equation (GEE).

Variable	VR group (*N* = 20)		Comparison group (*N* = 20)		Group effect	Time effect	Group × Time interaction
	MD	(95% CI)	MD	(95% CI)	*p*	*p*	*p*
*Physiological measures*							
Distress	−1.77	(−0.85, 1.57)	2.07	(−0.72, 4.86)	**0.021**	0.077	**0.015**
Depression	−1.08	(−5.17, 1.63)	0.50	(−0.51, 1.51)	**0.005**	0.470	0.062
Anxiety	−0.92	(−3.66, 1.50)	1.14	(−1.70, 3.99)	**0.039**	0.167	**0.042**
Somatization	0.85	(−2.55, 0.70)	1.71	(−1.44, 4.86)	0.340	0.062	0.452
Positive affect	0.22	(−1.36, 3.05)	−0.24	(−0.79, 0.31)	**0.035**	0.267	0.095
Negative affect	0.19	(−0.54, 0.99)	−0.21	(−0.57, 0.16)	0.518	0.371	0.297
Perceived stress	−0.71	(−2.40, 0.97)	0.86	(−1.27, 2.98)	0.475	0.315	0.116
*Physiological measures*							
SBP (mmHg)	0.88	(−5.91, 7.66)	9.73	(−0.45, 0.84)	0.357	0.590	**0.007**
DBP (mmHg)	0.13	(−7.65, 7.90)	2.47	(3.18, 16.29)	0.165	**0.031**	**<0.001**
HR (bpm)	−1.93	(−0.70, 5.63)	3.63	(−8.69, 15.94)	0.883	0.404	0.393
SDNN (ms)	6.59	(−5.98, 2.12)	−8.10	(−34.22, 18.02)	**0.030**	0.587	0.184
TP (Ln (ms^2^))	0.53	(0.13, 0.92)	−0.13	(−1.41, 1.15)	0.585	**0.011**	0.545
LF (Ln (ms^2^))	0.70	(0.15, 1.25)	−0.13	(−1.16, 0.89)	0.461	**0.001**	**0.041**
HF (Ln (ms^2^))	0.66	(0.03, 1.28)	−0.36	(−1.74, 1.02)	0.279	**0.006**	**0.028**
LF/HF (Ln (ratio))	0.05	(−0.44, 0.53)	0.23	(−0.40, 0.86)	0.473	0.534	0.933

The GEE was adjusted for participants’ age and score at the baseline. The statistical significance level is set at 0.05 and the value of statistical significance is emphasized in bold. VR, virtual reality; MD, mean differences between pre-and post-test scores; CI, confidence interval; SBP, systolic blood pressure; DBP, diastolic blood pressure; HR, heart rate; SDNN, standard deviation of all normal-to-normal intervals; TP, total power (0–0.5 Hz); LF, low-frequency power (0.04–0.15 Hz); HF, high-frequency power (0.15–0.40 Hz); LF/HF, the ratio of low frequency to high frequency.

For physiological measures, significant group effects (*p* = 0.031) and group-by-time interactions (*p* < 0.001) were found for DBP. A significant group-by-time interaction (*p* = 0.007) was found for SBP. HRV outcomes revealed that SDNN had a significant group effect (*p* = 0.030). Both LF (*p* = 0.041) and HF (*p* = 0.028) had significant group-by-time interactions. Mean differences indicated that the comparison group had higher blood pressure after 12 weeks. The SDNN, LF, and HF of the VR group had improved after the intervention compared to the comparison group. However, HR and LF/HF exhibited no significant effects.

## 4. Discussion

This study applied a VR device to bring the natural environment to furniture factory workers. VR natural experiences were introduced in the afternoon break time weekly for 12 weeks. Participants’ psychological stress, including distress, depression, anxiety, and positive affect, improved after long-term VR natural experiences. Participants’ physiological stress, including partial indicators of HRV and stabilized BP, improved after long-term exposure to VR natural experiences. VR natural experiences could potentially ameliorate factory workers’ stress levels.

This study found that VR natural experiences had positive effects on psychological stress of factory workers, including distress, depression, anxiety, and positive affect. These results are in line with previous studies and demonstrate the effects of VR natural experiences on alleviating psychological stress (Li et al., 2021; Calogiuri et al., 2022; Spangenberger et al., 2022) and that it is a feasible approach which can be applied during break time in the workplace, particular in manufacturing factory settings. However, VR natural experiences did not have an effect on perceived stress. A possible reason might be that other social stressors exist, such as job content, family situations, social dynamics among colleagues, and so on, that were potentially affecting perceived stress (World Health Organization, 2020). Both the work content and work context need to be assessed; for example, the work context includes career development, economic and payment issues, role in the organization, interpersonal relationships, organizational culture, and work-life balance (World Health Organization, 2020; Sanchez-Gomez et al., 2021). Future studies can consider evaluating other stressors to exclude stressed-out participants in order to examine the effect of VR natural experiences on perceived stress.

In addition, this study also found VR natural experiences had positive impacts on the physiological stress of factory workers, including the SDNN, LF, and HF. The SDNN is one of the important indicators of ANS functions which presents overall physiological stress measured by the HRV (Kim et al., 2018). On the other hand, participants in the experimental group had stable SBP and DBP after the intervention, while participants in the comparison group had increased SBP and DBP after 12 weeks. This is a relatively less explored area, and more biofeedback and physiological measures are encouraged to be managed by VR nature interventions. Our study adopted precise and accurate physiological measures in assessing ANS function with an evidence-based evaluation to summarize the effects of VR natural experiences (Francis et al., 2009; Guo et al., 2022). Study findings highlighted that changes in physiological outcomes can be achieved by applying a VR natural experience intervention in workplace settings which is central to promoting occupational health. As mentioned, VR natural experiences as a simulation-based intervention did contribute to psychological and physiological stress improvement. This study can be a fundamental work in the component of simulation of perception in the simulation theory. Future studies and interventions are warranted to investigate the impacts of the simulation-based intervention on other components of the simulation theory, namely simulation of behavior and anticipation (Hesslow, 2012). It is believes that the benefits of VR natural experiences can go beyond the psychological and physiological stress improvement, for example, the enhancement in working memory, motor, sensory and cognitive function may be observed as well. In our study, the VR natural experience intervention was only implemented during break time at a factory for 30 min per session. Thus, the frequency and length were reasonable and feasible, and did not occupy much time during their break. We suggest that employers could provide VR natural experience headsets in the break space or conference room in the factory so that factory workers can have options to relieve their tense mood and improve physiological stress during their break time and have a better occupational health status.

To our best knowledge, this is the first study to explore the effects of natural experiences *via* VR devices on psychological and physiological stress for occupational health. However, there were several limitations. The study design was a clustered RCT, instead of an RCT. A few variables of participant’s backgrounds significantly differed between the experimental and comparison groups, although most stress-related outcomes at the baseline did not differ between the two groups. The diverse population might have influenced the interpretation of results. The small sample size and loss of several participants after 12 weeks were also a problem. The 360° video might have been not clear enough compared to the real world. During the intervention, participants who complained about VR sickness might have missed several parts of the videos because they closed their eyes to rest. Finally, the activities of participants in the comparison group during the break time were not monitored, which might have generated a bias.

This study has implications for factory employers and occupational healthcare professionals such as public health nurses and general practitioners, and informs future studies for developing relevant interventions. Manufacturing industry employers should pay attention to employees’ occupational health, especially stress. Planning regular break time in relaxed and comfortable places is important for employees’ rest and further work efficacy. VR is an interesting platform that can provide an opportunity to connect with nature, activities, and games. Weekly real-world and VR-based natural experiences are both recommended for stressed workers. However, when using VR devices, the image resolution of 360° videos is still a problem that might cause VR sickness that should be overcome by future technological advances. For future studies, a study design of RCTs might provide stronger evidence. Increasing the sample size and decreasing differences in participants’ backgrounds between groups are important considerations. The intervention can be conducted more frequently and longer, for example, two or three times a week for 6 months. Diverse natural environments, such as forests, waterfalls, and mountains, can be recorded in a 360° format to increase enjoyment and attraction. Besides the visual and auditory stimulation from VR, olfactory stimulation could be considered in future interventions.

VR is an innovative opportunity to bring the natural environment into an indoor environment. VR natural experiences can provide similar effects as real-world natural experiences for relaxation. This study indicated that VR natural experiences had positive effects on furniture factory workers’ psychological and physiological stress after a 12 week intervention. VR natural experiences are recommended to release stress and promote the occupational health of factory workers and heavy labors.

## Data availability statement

The datasets generated during and analysed during the current study are available from the corresponding author on reasonable request.

## Ethics statement

The studies involving human participants were reviewed and approved by Taiwan Medical University-Joint Institutional Review Board (N202103114). The patients/participants provided their written informed consent to participate in this study.

## Author contributions

M-HH: formal analysis and writing—review and editing. M-SW: resources and data curation. H-YY: formal analysis, conceptualization, supervision, and writing—review and editing. All authors contributed to the article and approved the submitted version.

## Funding

This work was supported by the Ministry of Science and Technology, Taiwan under grant (MOST 109-2314-B-038-077-MY3).

## Conflict of interest

The authors declare that the research was conducted in the absence of any commercial or financial relationships that could be construed as a potential conflict of interest.

## Publisher’s note

All claims expressed in this article are solely those of the authors and do not necessarily represent those of their affiliated organizations, or those of the publisher, the editors and the reviewers. Any product that may be evaluated in this article, or claim that may be made by its manufacturer, is not guaranteed or endorsed by the publisher.

## Supplementary material

The Supplementary material for this article can be found online at: https://www.frontiersin.org/articles/10.3389/fpsyg.2023.993143/full#supplementary-material
