# Supplementary material for: Effects of virtual reality natural experiences on factory workers’ psychological and physiological stress
Source: Front Psychol. 2023 Mar 6;14:993143. doi: 10.3389/fpsyg.2023.993143 (PMC10025299; doi:10.3389/fpsyg.2023.993143)
Supplement: Supplementary file 1 [file Data_Sheet_1.pdf]

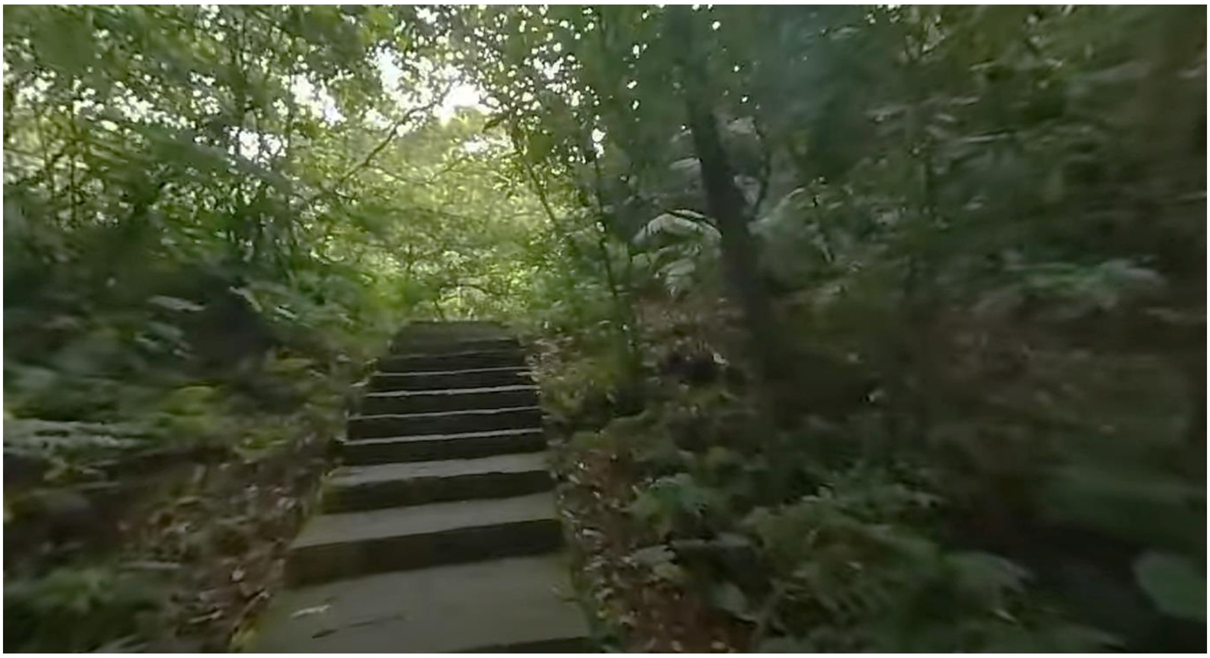

Supplementary Figure 1. The example scene of nature-based VR videos (the first week)

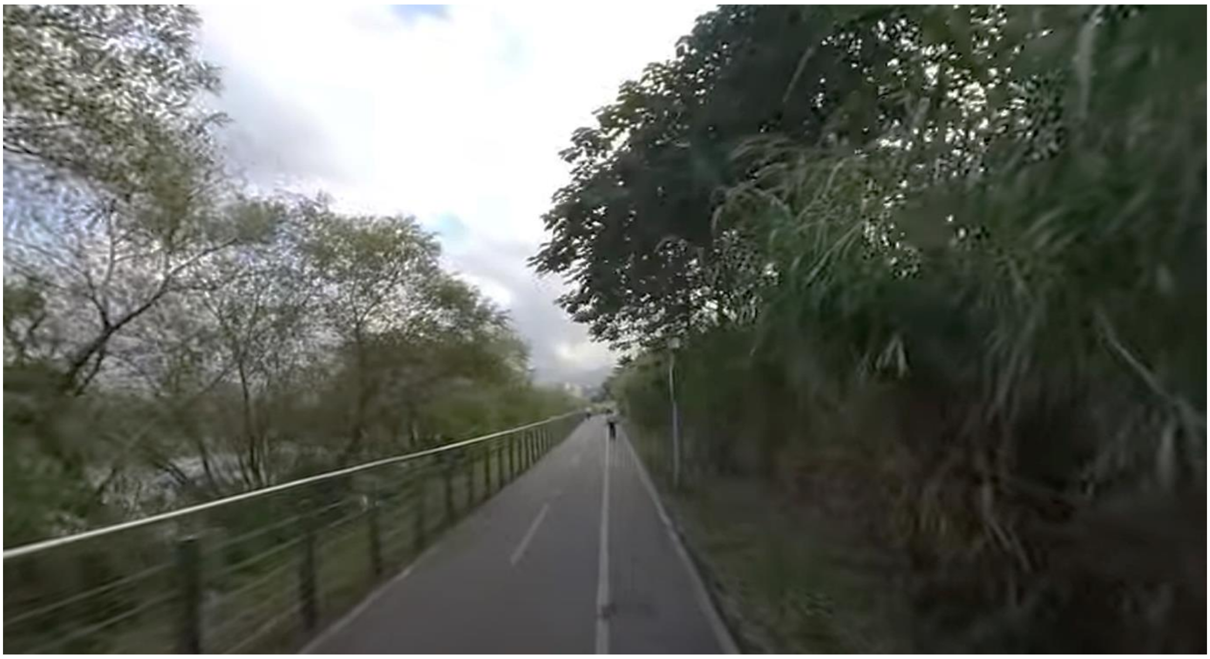

Supplementary Figure 2. The example scene of nature-based VR videos (the 11th week)
